# Real-Time Tool Detection for Workflow Identification in Open Cranial Vault Remodeling

**DOI:** 10.3390/e23070817

**Published:** 2021-06-26

**Authors:** Alicia Pose Díez de la Lastra, Lucía García-Duarte Sáenz, David García-Mato, Luis Hernández-Álvarez, Santiago Ochandiano, Javier Pascau

**Affiliations:** 1 Departamento de Bioingeniería e Ingeniería Aeroespacial, Universidad Carlos III de Madrid, 28911 Leganés, Spain; apose@ing.uc3m.es (A.P.D.d.l.L.); lucia.g.-@alumnos.uc3m.es (L.G.-D.S.); dgmato@ing.uc3m.es (D.G.-M.); 2 Instituto de Investigación Sanitaria Gregorio Marañón, 28007 Madrid, Spain; sochandiano@hotmail.com; 3 Departamento de Tecnologías de la Información y las Comunicaciones (TIC), Instituto de Tecnologías Físicas y de la Información (ITEFI), Consejo Superior de Investigaciones Científicas (CSIC), 28006 Madrid, Spain; luis.hdez.alvarez@iec.csic.es; 4 Servicio de Cirugía Oral y Maxilofacial, Hospital General Universitario Gregorio Marañón, 28007 Madrid, Spain

**Keywords:** Artificial Intelligence, deep learning, craniosynostosis surgery, phase estimation, tool detection

## Abstract

Deep learning is a recent technology that has shown excellent capabilities for recognition and identification tasks. This study applies these techniques in open cranial vault remodeling surgeries performed to correct craniosynostosis. The objective was to automatically recognize surgical tools in real-time and estimate the surgical phase based on those predictions. For this purpose, we implemented, trained, and tested three algorithms based on previously proposed Convolutional Neural Network architectures (VGG16, MobileNetV2, and InceptionV3) and one new architecture with fewer parameters (CranioNet). A novel 3D Slicer module was specifically developed to implement these networks and recognize surgical tools in real time via video streaming. The training and test data were acquired during a surgical simulation using a 3D printed patient-based realistic phantom of an infant’s head. The results showed that CranioNet presents the lowest accuracy for tool recognition (93.4%), while the highest accuracy is achieved by the MobileNetV2 model (99.6%), followed by VGG16 and InceptionV3 (98.8% and 97.2%, respectively). Regarding phase detection, InceptionV3 and VGG16 obtained the best results (94.5% and 94.4%), whereas MobileNetV2 and CranioNet presented worse values (91.1% and 89.8%). Our results prove the feasibility of applying deep learning architectures for real-time tool detection and phase estimation in craniosynostosis surgeries.

## 1. Introduction

In recent years, Artificial Intelligence (AI) has revolutionized the research landscape in healthcare, improving tasks such as genomic analysis, medical imaging, robot-assisted surgery, and natural language processing [1]. Deep learning (DL) is a subfield of AI that has introduced the architectures called Convolutional Neural Networks (CNNs). These solutions have surpassed human performance in image processing and classification, although their introduction in the clinical workflow is still a challenge [2]. One example is the tracking of laparoscopic instruments during minimally invasive surgeries. This problem was addressed by Zhao et al. combining a CNN with spatio-temporal information [3], achieving better performance than mainstream detection methods. Jin et al. showed how tool detection with VGG16, a CNN architecture, allowed the calculation of metrics such as range of motion and tool usage time [4], while other methods combined this CNN with heat maps [5].

If tool detection algorithms provide good results, the next step is the automatic recognition of surgical workflows. Identifying different phases in a surgical procedure could be beneficial in many aspects: on the one hand, it can facilitate intraoperative support, providing automated assistance and objective feedback [6]; moreover, real-time warnings can be displayed when unexpected workflow variations or adverse events are detected, reducing the rate of complications in operating rooms (OR) and enhancing patient’s safety [7]. Measuring the actual time of each surgical step may also improve communication and coordination of the clinical staff, increasing the hospital’s efficiency [8]. On the other hand, the surgeons’ expertise influences the post-operative results [4]: novel surgeons are more prone to errors in the OR. In fact, those students that have trained with simulators tend to perform better than those that followed traditional learning [9]. Consequently, contextual support may guide junior clinicians during their first interventions to increase their confidence and enhance their outcomes.

In this context, Jin et al. [10] developed a multi-task recurrent convolutional neural network (R-CNN) for surgical tool detection and phase recognition in minimally invasive surgery. Both tasks were also studied in [11] with an AlexNet-derived network (namely, EndoNet) for laparoscopic surgeries. They used ensembling techniques to combine the probabilities of two different models, improving the classifier. Finally, [12] presented an InceptionV3 network to classify cataract surgical phases with temporal information.

Despite the number of surgical procedures in which AI has improved workflow analysis, up to our knowledge, no studies have applied these techniques to open cranial vault remodeling for the correction of craniosynostosis. Craniosynostosis is a congenital defect that implies the premature fusion of one or more cranial sutures that separate the skull bones [13]. This condition may be induced by genetic, teratogenic, or mechanical causes or can even arise sporadically [14]. It affects one in every 2000 to 2500 live births [15] and produces cranial deformities that may limit brain growth. Considering that a newborn brain quadruples its size during the first year of life [16,17], this defect can result in very important functional and structural alterations. To protect the child from physical and mental disorders, it is crucial to correct the malformations on time [18]. The standard treatment consists of a surgical intervention that remodels the affected bone tissue into the most appropriate shape for the patient. Open cranial vault remodeling is preferably performed before the first year of life to benefit from the malleability of bone tissue [19].

However, this complex procedure depends highly on the surgeon’s subjective judgment, knowledge, and previous experience [20]. Thus, the incorporation of novel technologies to guide surgeons during these interventions is crucial to guarantee reproducibility and efficiency [21]. To objectify the intervention and ensure that the surgical outcomes followed the preoperative planning, our group defined the steps for an optimized surgical workflow [16]. The process included designing and 3D printing of surgical guides and patient-specific templates. Furthermore, we used real-time navigation with an optical tracking system (OTS) to guide the placement of the remodeled bones during the surgical intervention. The method showed positive results in five patients.

We propose this research as a proof-of-concept to test the possibility of applying DL algorithms to further improve craniosynostosis interventions. Specifically, we developed a new method for the automatic and real-time estimation of the craniosynostosis surgical workflow based on surgical tool detection. In the long term, this could facilitate intra-operative assessment and post-operative analysis. Moreover, the application of these methods during surgical training could improve the proficiency of medical students, shorten their learning curve, and reduce inter-surgeon variability. To our knowledge, this is the first study of automatic tool detection for craniosynostosis surgical procedures. The detection phases defined in this work are a simplification of those proposed in [16]. We have developed four DL algorithms (based on different CNN architectures) integrated in a novel 3D Slicer module to achieve these goals. Surgical tools present in live or recorded video streams can be automatically identified in real-time. The system was tested during a simulation of a craniosynostosis surgery, including nine distinct tools, on a realistic phantom created from an infant’s head as described in [17].

## 2. Materials and Methods

The proposed solution for phase recognition in craniosynostosis surgery is detailed in the following subsections. First, we describe the tools to be recognized and the phases under study (Section 2.1). The data acquisition process and the different datasets are presented in Section 2.2. Section 2.3 contains information about the network architectures evaluated, while Section 2.4 describes the training process and parameters and Section 2.5 the phase estimation method. Finally, Section 2.6 explains the evaluation protocol and metrics.

### 2.1. Experimental Set Up

We simulated in the laboratory an open cranial vault remodeling procedure with fronto-orbital advancement for the treatment of metopic craniosynostosis using the 3D printed patient-specific phantom described in [17]. This phantom is based on a patient that suffered from severe trigonocephaly malformation (fusion of the metopic suture), and consists of a head with skull, brain, and skin. The bone was 3D printed in polylactic acid (PLA) using a fused deposition modeling (FDM) desktop 3D printer (Ultimaker B.V., Utrecht, Netherlands). Soft tissue was simulated with silicone.

**Figure 1:**
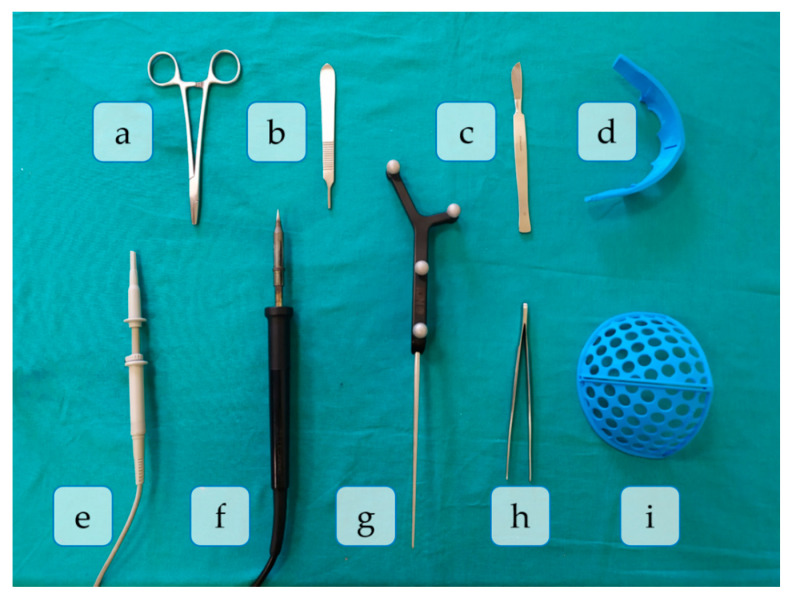
Surgical tools used in the study. (**a**) Scissors; (**b**) Osteotome, (**c**) Scalpel, (**d**) SO guide, (**e**) Motor, (**f**) Handpiece, (**g**) Pointer, (**h**) Forceps, (**i**) FT guide.

The surgical procedure was divided into eleven different phases, according to the activity conducted and tools used in each one. A total of nine tools were necessary for this surgical simulation, namely: scalpel knife (scalpel), osteotome emulator (osteotome), claw dissection forceps (forceps), supraorbital surgical cutting guide (SO guide), frontal surgical cutting guide (FT guide), emulator of a SonicWeld Rx (handpiece), tracked tool (pointer), emulator of a surgical ultrasonic bone-cutting device (motor) and scissors. All these tools are depicted in Figure 1 and employed in each surgical phase as explained below:

P1. Skin incision: A bicoronal S-shaped incision is performed over the phantom’s skin using the scalpel.
P2. Cranial surface exposure: The skin is kindly removed towards the anterior part of the head until completely revealing the cranium. Osteotome and forceps are used in this step.
P3. Surgical cutting guides placement: the SO guide and the FT guide are placed on the supraorbital and frontal regions of the skull, respectively.
P4. Fixation of registration pins: both surgical guides are fixed to the bone using surgical pins. In this phase, the SO guide, the FT guide, and the handpiece are present in the surgical field.
P5. Registration: the OTS and the tracked tool (pointer) are used to record the position of reference points on the surgical guides for intraoperative registration. Both surgical guides can also be identified in the image.
P6. Frontal bone osteotomy: after removing the surgical guides, the frontal bone is cut with the motor.
P7. SO bar bone osteotomy: the motor is now used to cut the SO bar bone with the aid of the osteotome, to avoid damaging the brain.
P8. Intraoperative navigation of remodeled bones: the positions of the remodeled SO bar and frontals bones are navigated with the pointer once they are placed back on the patient. This data is then compared with the preoperative surgical plan to ensure correct placement of the fragments.
P9. Screwing: the remodeled bone fragments are fixed and stabilized using resorbable plates and pins. Pins are welded to the adjacent bone tissue using the handpiece. Forceps hold the resorbable plates in place while pins are inserted.
P10. Skin placement: with the aid of the forceps, the skin is repositioned.
P11. Suturing: the skin is sutured with surgical scissors and forceps.

**Figure 2:**
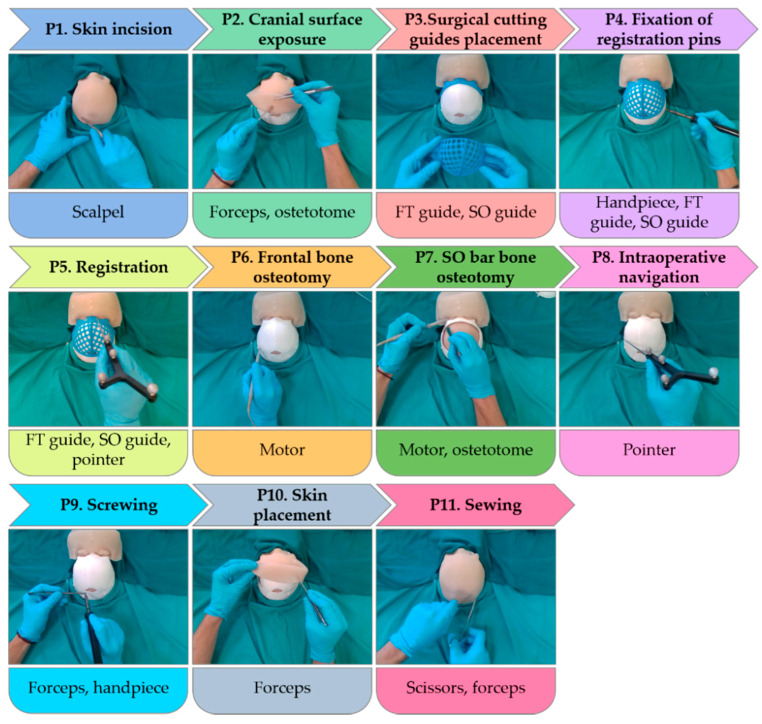
Examples of testing dataset frames for each surgical phase.

Figure 2 shows some examples of the surgical field appearance during the phases of the surgical simulation and the tools involved in each step.

### 2.2. Data Acquisition

We recorded different video streams of roughly one minute duration at a rate of 15 frames per s with 3D Slicer software (sequences module). All the recordings were made over a table with a green surgical sheet to resemble a clinical scenario. The camera (Intel RealSense D415 from Intel Corporation, Santa Clara, CA, USA) was positioned on top of the simulated surgical field to approximate the surgeon’s perspective during the intervention. We collected two different datasets for training (Training dataset) and testing (Testing dataset) purposes. In both cases, we moved none, one, or multiple surgical tools in front of the camera, held by one or two hands with blue latex gloves.

- Training dataset: This included roughly 10,000 frames obtained from thirteen video streams. Eleven of these contained the combinations of tools appearing in the surgical phases, with the phantom and the surgical sheet in the background. However, instead of replicating the surgical procedure, we showed the tools from different perspectives and varying positions, partially covering them repeatedly with the hand (or other tools) or even taking them out from the FOV to a certain extent. The other two videos recorded the empty hands and the simulated patient individually over the green surgical sheet background. Only in the latter case, we manipulated the phantom to show the skin, the skull, the bone fragments, and the brain, keeping it static in the other videos. The surgical sheet was wrinkled differently in all video streams to teach the model to ignore the background and only focus on the foreground. Moreover, we acquired the videos under varying illumination conditions: natural light, artificial light, and moving shadows.
- Testing dataset: This was obtained from a single video of roughly six minutes duration recorded during a fast simulation of a complete craniosynostosis surgery. This video was recorded on a different day and by another user, repeating the experiment set up from scratch. As a result, this video’s composition differed sufficiently from the training recordings to prevent overfitting. The illumination conditions were changed several times per phase during the video recording to test the system’s robustness against varying lighting circumstances. As in the training videos, we worked with natural light, ambient light, and moving shadows. We extracted all the frames of that video (4920) and labeled them to calculate the evaluation metrics described in Section 2.6. Furthermore, the same video was directly streamed in a specifically developed 3D Slicer module to predict, in real-time, the tools that appeared on each frame (see Section 2.5).

### 2.3. Deep Learning Networks

We trained a total of four networks. The first one is a simple, novel, and customized CNN (CranioNet) specifically developed for this study. It consists of a sequence of blocks made up of two 3 × 3 convolutions with a stride of 1, followed by a 3 × 3 max pooling operation with a stride of 2. reLU activation function and batch normalization are applied after each convolution. Dropout is then introduced with a probability of 0.25. There is no padding except for the convolutions of the first block (P = 1). Next, two fully connected (dense) layers with a reLU activation, a batch normalization, and a dropout with 0.5 between them are performed. The output layer (second dense layers) is a Sigmoid classifier.

We selected the following parameters for the training process: binary cross-entropy loss function with weighted L1 regularization; Adam optimizer; and initial learning rate α=0.0001. The remaining hyperparameters were set with the Keras default values: decay constant β=0.9, and $\epsilon =10^{-8}$. We used Xavier initialization for the weights and zero initialization for the biases. The total number of trainable parameters was 3,568,714. Images were passed through the network in batches of 64. The model was trained for 20 epochs.

The remaining CNNs are three state-of-the-art networks: VGG16, MobileNetV2, and InceptionV3. They were all trained with transfer learning. VGG architectures include more convolutional layers to improve classification accuracy. However, these convolutional filters are very small, decreasing the number of parameters and increasing non-linearities to make the classification process more discriminative [22]. The main limitation of these networks is the high computational cost due to their depth. Inception networks have aggressive dimension reductions that decrease computational burden while retaining accuracy. Besides, they provide high-quality results with low-resolution images, which is useful when recognizing small objects [23]. MobileNet models maintain quality while reducing computational costs by introducing depthwise separable convolutions [24]. They improve performance by decreasing the number of operations and removing non-linearities from their narrower layers [25].

Transfer learning was implemented by replacing the last layers of the base models with a global average pooling (spatial averaging while keeping depth dimensions), followed by a 0.5 probability dropout and a Sigmoid classifier for all the labels. The parameters of the base models were initialized with those pre-trained on ImageNet [26] and frozen so that they did not update during training. Only the parameters of the newly introduced layers were trained, for instance: 37,670,922 parameters for VGG16, 42,979,338 parameters for InceptionV3 and 40,816,650 parameters for MobileNetV2. All networks were trained using Adam optimizer, β=0.9, and $\epsilon =10^{-8}$. However, the learning rate was tuned and fixed at 0.00001. The batch size remained the same (64), while the number of epochs was reduced to 10.

### 2.4. Training of the Networks

For the tool recognition task, we propose a multilabel problem in which every tool (and the environment) corresponds to a label. The environment label was assigned to every no-tool element, such as the patient or the empty hands. We extracted the frames from the videos and assigned labels according to the tools appearing on them. For instance, we stored all the frames from a video stream showing the forceps and the osteotome with the phantom in the background in a folder named “Environment_Forceps_Osteotome”. Then, a one-hot-encoder vector was automatically assigned to each frame, with 1s in the positions of the visible tools and 0s elsewhere. Once uploaded, the images were mixed up to start the training process. The sigmoid function in the output layer of all the models enabled us to treat each label independently. The train:validation sets were distributed in a proportion of 80:20, corresponding to 7947:1987 images of the training dataset.

Input images were resized to 64 × 64 spatially and pre-processed for data normalization. We evaluated the loss functions during the training and validation of all the models to ensure convergence. We also performed data augmentation with the ImageDataGenerator API from Keras to prevent overfitting. This method provides real-time data augmentation, decreasing memory usage. Image transformation parameters are summarized inTable 1.

### 2.5. Phase Estimation

Once the models were trained, they were exported to our 3D Slicer module (version 4.11.20200930) [27,28]. This module was created to test the accuracy of the CNN models with the testing dataset. The video stream of the surgical simulation was loaded in the module, which executes the models to identify and display the tools appearing in the images while the video is played. This module can also be connected to a camera to classify the input video stream in real-time. Live and recorded videos can be streamed into the software application using the OpenIGTLink communication protocol [29]. The confidence to determine if a tool is present or not is measured from 0 (the tool is not present at all) to 1 (the tool is present with 100% certainty). To consider a tool as detected we selected a threshold of 0.5. If no tool probability is above the threshold, the word “None” is shown. Once the process stops, all temporal and classification data can be stored in a CSV file for further analysis.

**Figure 3:**
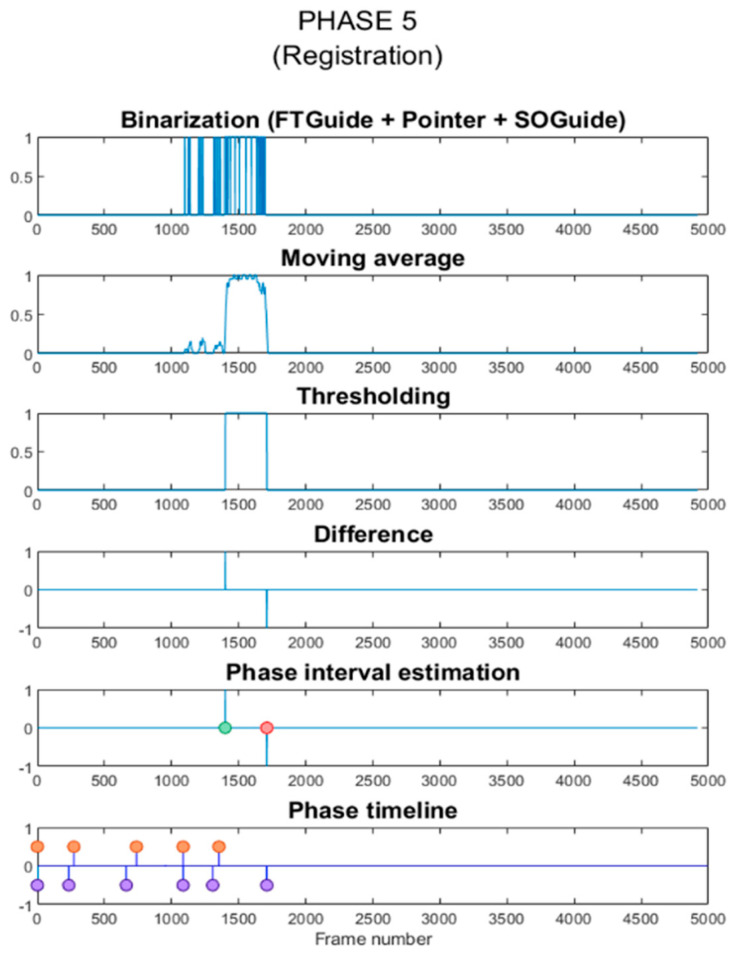
Graphical representation of the postprocessing pipeline for phase estimation.

Each workflow phase is defined by the tools present in the surgical field at that moment (right-phase tools). We analyzed the CSV file and created a temporal binary signal with 1s when the right-phase tools were detected and 0s when there were other tools in the FOV (wrong-phase tools). We then applied a moving average filter to smooth the signal, thresholding the result to obtain binarized data. A difference vector is calculated to detect the beginning and end of the batches of 1s. Temporal information was used to identify the beginning of the phase. This means that every new phase must start after the end of the previous phase. Consequently, hits before that time are automatically discarded. The duration of the phase was determined by the length of the first batch of 1s after that moment. The interphases’ limits are estimated averaging the end peak of the previous phase and the start peak of the new one. This process is represented in Figure 3, on which P5 is estimated from the results obtained using the InceptionV3 model.

### 2.6. Performance Evaluation

The performance of the networks was evaluated in terms of tool classification precision (Equation (1)), recall (Equation (2)), F1 score (Equation (3)), and accuracy (Equation (4)), drawn from the true positives (TP), true negatives (TN), false positives (FP), and false negatives (FN) [2]. The first three metrics were used to study the ability of each model to detect each individual tool. In this sense, the precision can be understood as the quality of the model (percentage of tools correctly identified), while the recall represents the number of tools identified. Since the F1 score is the harmonic mean of these two metrics, it facilitates comparison of the combined performance of precision and recall among different solutions.

(1) $$Precision=\frac{TP}{TP+FP}$$

(2) $$Recall=\frac{TP}{TP+FN}$$

(3) $$F1\;score=2\times \frac{Precision\times Recall}{Precision+Recall}$$

(4) $$Accuracy=\frac{TP+TN}{TP+FP+TN+FN}$$

In complementary fashion, the accuracy represents the percentage of frames that have been correctly identified. In other words, it is the percentage of images in which the tools recognized by the network are precisely and uniquely the right-phase tools of the frame. This nuance is essential, as an image that contains three tools will be misclassified if the model recognizes four tools. However, only the precision, recall, and F1-score of the fourth tool would worsen, as a TP or TN is obtained for every tool except for that one (for which a FP is obtained).

Regarding the detection of the different surgical phases, the performance of the model was analyzed by comparing the start and end times of the ground truth and predicted phases. In this way, if a phase was predicted to start before and end after its corresponding ground truth, it was considered that 100% of the phase was detected. When the predicted phase started after or ended before the real phase, the performance dropped, as the complete phase was not identified.

## 3. Results

### 3.1. Tool Detection

The training and inference times of each CNN are collected in Table 2. Precision, recall, F1-score, and accuracy metrics (in percentage) obtained for each model are presented in Table 3.

Despite the simplicity of CranioNet, it needed roughly the same time per epoch as its partners to be trained. In fact, since it was trained with 20 epochs, the total time required to obtain results was approximately double. Nevertheless, after training, the inference time of this network is roughly half of the other three. With respect to the models’ accuracy, CranioNet presents the lowest outcomes for tool recognition: 93.4%. The highest accuracy is achieved by the MobileNetV2 model (99.6%), followed by VGG16 and InceptionV3 (98.8% and 97.2%, respectively). Regarding the individual tool metrics, the results show that the FT guide and the scissors are perfectly identified by all the models, probably due to the distinctive shape of both tools. The remaining tools also present remarkable values for their precision, recall, and F1-score, except for the motor in CranioNet. In that case, the precision and F1-score are, respectively, 72.5% and 84.1%. The difficulties of this model identifying the motor are probably the leading cause for its low accuracy.

To illustrate these results better, Table 4 depicts the percentage of TP, TN, FP and FN obtained for each tool with the InceptionV3 model. Additionally, we show the differences in the detection of the motor in Table 5. It should be noticed that the number of times each individual tool is present in the dataset is lower than the sum of appearances of the rest of the tools. That is, for all tools, the number of TP is considerably lower than the number of TN. As a result, the number of FP represents a low percentage in comparison with the number of TN. Still, it has a more significant impact on the precision calculation, as it is compared with the number of TP.

### 3.2. Phase Detection

**Figure 4:**
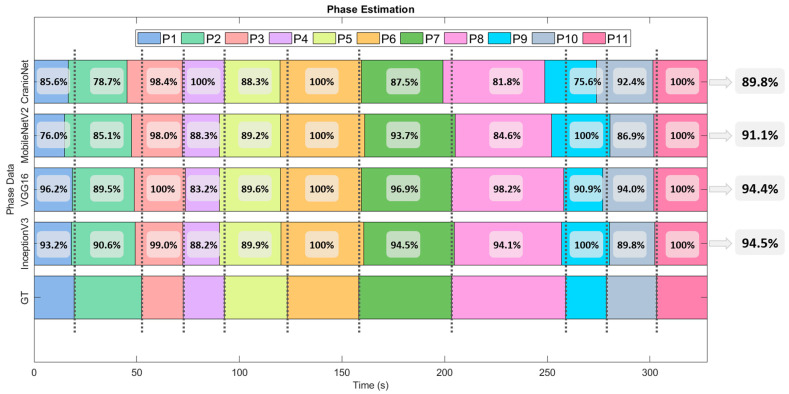
Individual phase estimation for the testing dataset using CranioNet, MobileNetV2, VGG16 and InceptionV3 DL models. Classification accuracy for each phase and model is shown for each interval. The average classification accuracy of each model is displayed on the right.

Figure 4 shows the percentage of ground truth phase detected for each individual phase and model. InceptionV3 and VGG16 achieve the best results, with an average phase detection of 94.5% and 94.4%. MobileNetV2 achieves a result of 91.1% and CranioNet 89.8%.

## 4. Discussion

The benefits of automatic tool detection and phase estimation for different medical applications have been demonstrated in previous studies [4,5,7,9,30]. However, none of these analyzed these solutions in the context of open cranial vault remodeling for craniosynostosis correction. In this work, we propose AI-based algorithms to improve the efficiency and reproducibility of craniosynostosis interventions. Specifically, we have developed four deep learning networks capable of recognizing surgical tools, in real-time, using a video camera and a specific application developed on 3D Slicer. Our main goal was to offer real-time, helpful guidance on craniosynostosis surgeries with an easy-to-use system that could be deployed to clinical users at a low cost. With this solution, we could obtain statistics related to tool usage and phase duration that would benefit surgeons during their training for these interventions.

Incorporating context-aware software in the OR would provide accurate feedback and warnings when unexpected variations are detected during the workflow. Besides, the information displayed during surgical navigation could be adapted to the needs of the current phase, improving surgeon experience. On the other hand, the system can also evaluate novel surgeons, provide the duration of each phase, assess the correct tool utilization, and improve surgeons’ confidence in the workflow. Overall, incorporating this novel technology in the craniosynostosis surgical workflow will enhance the efficiency and reproducibility of these interventions, reducing surgical errors and complications. This, in turn, could imply an enhancement in efficiency and surgical outcomes.

This study has improved the methods and extended the experiments from our previous work presented in [31]. In this case, the system includes the recognition of multiple simultaneous tools independently. This new feature enables us to identify the surgical phases better since several tools are commonly present in the surgical field at a time. On the other hand, in this study, all the experiments were performed under different illumination conditions. We tested the system during the simulation of a craniosynostosis intervention on a realistic phantom, and it showed promising results for both tasks despite these variations. This suggests that our system is robust against this feature and, therefore, illumination independent.

The results on tool classification indicate that, despite the differences between VGG16, InceptionV3, and MobileNetV2, they all seem to be good choices for tool identification. There is a reduction in accuracy if we compare these networks with CranioNet. Despite this, it contains more than ten times fewer parameters than the other networks, making it suitable for deployment to less powerful devices (such as smartphones or augmented reality glasses) in future applications. We did not train CranioNet with transfer learning, but we built it from scratch and trained it only with craniosynostosis simulation images. Finally, although CranioNet training time doubles those from other networks, tool detection speed during inference is two times faster than the alternatives. In fact, processing frames at 0.033 s/frame is the standard for real-time videos and CranioNet inference time was of 0.036 s/frame. Considering these advantages, the slight drop in accuracy seems a fair price to pay, as we are interested in a real-time system.

Regarding phase estimation, CranioNet has proven to perform worse than the others. Despite this, the results obtained with this net are already comparable to the state-of-the-art in similar DL applications [6,8]. Interestingly, the results of the MobileNetV2 network, which produced the best accuracy in tool detection, are worse than those of InceptionV3 and VGG16. This suggests that, although MobileNetV2 classified more images correctly, those in which it failed were critical points of the surgery, like the frames in which the tools were exchanged. As an example, looking in detail at the predictions of phases P1 and P2 (the ones with the lowest overall performance), MobileNetV2 incorrectly detected the presence of a motor in the last frames of P1. Regarding the other three models, the performance is affected by confusing the scalpel with the osteotome (characteristic tools of P1 and P2, respectively), which have similar appearances.

Our system presents some limitations. The networks were trained with images of a single tool for each label. To extend our results to other surgical interventions, training images should include different variations of the same tools (such as other brands, shapes, or colors). Moreover, we could add more “no-tool” or “environment” elements in the surgical field, such as another surgeon, blood, or wires. Along the same line of thought, it would also be beneficial to make all the acquisitions in a real surgical scenario, to have more reliable light conditions and an actual patient in the field of view. Finally, our research could improve by identifying the tool position within the surgical field using a bounding box.

In conclusion, we have shown the feasibility of applying deep learning architectures for real-time tool detection and phase estimation in craniosynostosis surgeries. We believe that this work presents a reference for future studies that use AI to strengthen the outcomes in this clinical area.

## Figures and Tables

**Table 1:** Transformation parameters for image augmentation in training and validation data.

Methods	Range
Rotation angle	±15 pixels
Width shift	±10%
Height shift	±10%
Shear	±50%
Zoom	±10%
Horizontal flip	Enabled

**Table 2:** Training and inference times for each CNN using GPU.

CNN	Number of Epochs	Training Time per Epoch (s)	Total Training Time (min)	Inference Time (s/Frame)
CranioNet	20	79.6	26.55	0.036
MobileNetV2	10	69.8	11.63	0.054
VGG16	10	80.7	13.45	0.053
InceptionV3	10	76.2	12.70	0.059

**Table 3:** Precision (P), recall (R), F1 score (F1), and accuracy (Acc) for all the tools obtained by each network.

Tool	CranioNet				MobileNetV2				VGG16				InceptionV3			
	%P	%R	%F1	%Acc	%P	%R	%F1	%Acc	%P	%R	%F1	%Acc	%P	%R	%F1	%Acc
Forceps	100	100	100	93.4	100	100	100	99.6	99.3	100	100	98.8	97.8	100	98.9	97.2
FT guide	100	100	100		100	100	100		100	100	100		100	100	100	
Handpiece	95.8	100	97.9		100	100	100		97.1	100	98.5		100	94.2	97.0	
Motor	72.5	100	84.1		98.5	100	99.2		100	100	100		98.5	97.0	97.7	
Pointer	100	95.1	97.5		100	99.0	99.5		100	100	100		98.1	99.0	98.5	
Osteotome	94.5	100	97.1		100	100	100		100	97.2	98.6		98.5	97.1	97.8	
Scalpel	100	100	100		100	100	100		96.4	100	98.2		100	100	100	
Scissors	100	100	100		100	100	100		100	100	100		100	100	100	
SO guide	100	100	100		100	100	100		100	100	100		100	99.3	99.6	
Average	95.9	99.5	97.4		99.8	99.9	99.9		99.2	99.7	99.5		99.2	98.5	98.8	

**Table 4:** Classification accuracy of InceptionV3 DL model for all the tools in terms of percentages of true positives (TP), true negatives (TN), false positives (FP) and false negatives (FN).

Tool	% True Positives (TP)	% True Negatives (TN)	% False Positives (FP)	% False Negatives (FN)
Forceps	100	99	1	0
FT guide	100	100	0	0
Handpiece	94	100	0	6
Motor	97	100	0	3
Pointer	99	100	0	1
Osteotome	97	100	0	3
Scalpel	100	100	0	0
Scissors	100	100	0	0
SO guide	99	100	0	1

**Table 5:** Classification accuracy of CranioNet, MobileNetV2, VGG16 and InceptionV3 DL models for the motor tool in terms of percentages of true positives (TP), true negatives (TN), false positives (FP) and false negatives (FN).

CNN.	% True Positives (TP)	% True Negatives (TN)	% False Positives (FP)	% False Negatives (FN)
CranioNet	100	94	6	0
MobileNetV2	100	100	0	0
VGG16	100	100	0	0
InceptionV3	97	100	0	3

## Data Availability

Not applicable.
